# Making radiation therapy more effective in the era of precision medicine

**DOI:** 10.1093/pcmedi/pbaa038

**Published:** 2020-12-01

**Authors:** Xingchen Peng, Zhigong Wei, Leo E Gerweck

**Affiliations:** Department of Radiation Oncology, Massachusetts General Hospital, Harvard Medical School, Boston, MA 02114, USA; Department of Biotherapy, Cancer Center, West China Hospital, Sichuan University, Chengdu 610041, China; Department of Radiation Oncology, Massachusetts General Hospital, Harvard Medical School, Boston, MA 02114, USA

**Keywords:** radiation therapy, conventional fractionation, hypofractionation, cancer stem cell, cancer treatment

## Abstract

Cancer has become a leading cause of death and constitutes an enormous burden worldwide. Radiation is a principle treatment modality used alone or in combination with other forms of therapy, with 50%–70% of cancer patients receiving radiotherapy at some point during their illness. It has been suggested that traditional radiotherapy (daily fractions of approximately 1.8–2 Gy over several weeks) might select for radioresistant tumor cell sub-populations, which, if not sterilized, give rise to local treatment failure and distant metastases. Thus, the challenge is to develop treatment strategies and schedules to eradicate the resistant subpopulation of tumorigenic cells rather than the predominant sensitive tumor cell population. With continued technological advances including enhanced conformal treatment technology, radiation oncologists can increasingly maximize the dose to tumors while sparing adjacent normal tissues, to limit toxicity and damage to the latter. Increased dose conformality also facilitates changes in treatment schedules, such as changes in dose per treatment fraction and number of treatment fractions, to enhance the therapeutic ratio. For example, the recently developed large dose per fraction treatment schedules (hypofractionation) have shown clinical advantage over conventional treatment schedules in some tumor types. Experimental studies suggest that following large acute doses of radiation, recurrent tumors, presumably sustained by the most resistant tumor cell populations, may in fact be equally or more radiation sensitive than the primary tumor. In this review, we summarize the related advances in radiotherapy, including the increasing understanding of the molecular mechanisms of radioresistance, and the targeting of these mechanisms with potent small molecule inhibitors, which may selectively sensitize tumor cells to radiation.

## Introduction

Cancer is a primary health problem worldwide. Radiotherapy is one of the most common treatment modalities, with 50%–70% of cancer patients receiving radiation during the course of their illness.^1–3^ Given the radiobiological differences between tumors and normal tissues, such as those in the proliferative rate and the dose-response relationship, and the relatively rapid decrease in beam intensity with depth in tissue, traditional radiotherapy has for decades been administered as daily fractions of approximately 1.8–2 Gy given over several weeks. With this treatment schedule, although positive and improved treatment outcomes have been achieved, a considerable portion of patients suffer local recurrence.^4^ Radiation associated technological advances (e.g. increases in beam energy, particle irradiation, both of which can be exploited to enhance beam conformality, and advances in imaging techniques and planning systems) have promoted the evolution of radiation therapy into a precise treatment modality, which permits the administration of larger doses to tumors while sparing adjacent normal tissues.^5^ These technological advances have also reduced constraints on the development or consideration of unconventional treatment schedules. In relatively recent studies, hypofractionated (large dose per fraction) radiation has shown clinical advantage over conventional fractionated radiation for some tumor types.^6–8^ Hypofractionation like stereotactic body radiotherapy (SBRT), using accurate delivery of high doses to the tumor in a few fractions, decreases the dose and toxicity to neighbouring normal tissues.^9^ Meanwhile, hypofractionation reduces the frequency and number of radiotherapy sessions, with significant potential for a reduction in overall treatment time and cost. In addition to these clinical results, extant experimental studies suggest that large but sub-curative doses of radiation may render recurrent tumors sensitive to subsequent radiation.^10,11^ In this review, we discuss molecular mechanisms involved in radioresistance and possibilities for enhancing therapeutic efficacy in the era of precision radiation therapy.

## Do putative cancer stem cell markers reliably identify cancer stem cells (CSCs)?

Cancer is a heterogeneous disease with significant inter- and intra-tumoral diversity,^12,13^ with many studies showing that only a small fraction of tumor cells exhibit tumor initiating capability.^14,15^ Tumor initiating and sustaining cells which exhibit multilineage differentiation^16–18^ are referred to as cancer stem cells, although any tumor cell exhibiting a lack of normal growth control, i.e. sustained and unlimited reproductive capacity with invasive properties, may be considered a cancer initiating or sustaining cell. Researchers have identified many specific cancer stem cell markers, which are used to isolate or identify cancer stem cells within the bulk tumor cell population. Most but not all markers function in cellular attachment. For example, in melanoma, cancer stem cell markers include ABCB5, ALDH1, CD20, CD133 and CD271.^19,20^ For lung cancer, reported stem cell markers include ABCG2, ALDH1, CD90, CD117 and CD133.^21^ Breast stem cell markers include ALDH1, CD24, CD44, CD90, CD133 and α_6_–integrin.^22^ Reported colon cancer stem cell markers include ABCB5, ALDH1, β-catenin, CD24, CD26, CD29, CD44, CD133, CD166 and LGR5.^23^ However, these markers must be utilized with caution, i.e. they do not necessarily identify all cancer stem cells or any specific one. For example, CD44 was reported to be a specific breast cancer stem cell marker, but CD44 has diverse splice variants. Full-length CD44 was thought to be an ideal stem cell marker,^24,25^ but recently, the CD44v6 splice variant has been reported to be a more specific marker.^26,27^ In addition, some cancer stem cell markers are derived from mouse tumor cells and have not been validated in human samples.^20^ Thus, it is unclear whether these markers can be used to identify all human cancer initiating and sustaining cells. Cancer stem cell markers may be lost during the self-renewal process. CD133, a lung cancer and glioma stem cell marker, is sometimes inactivated in both tumor types because of CpG island methylation.^28^ Thus, CD133 may not be expressed in all lung or glioma tumors or may not be uniformly expressed in all sections of the same tumor. From these studies, it may be concluded that a single cancer stem cell marker is not uniquely or invariantly specific and generally should be used in combination with others. An additional consideration pertains to the various methods which can or must be used to identify different markers.^29^ Some markers can only be detected by flow cytometry while others can only be examined by immunohistochemistry. A single method may not be capable of detecting all putative markers within the same tumor.

A second prominent characteristic of cancer stem cells is their dynamic nature. Cancer stem or tumor initiating cells are validated by their ability to initiate tumors in immune deprived hosts. Studies performed in the 1970s demonstrated that the number of transplanted tumor cells needed to initiate tumors in syngeneic or immune compromised mice may markedly decrease by co-injection of lethally irradiated “feeder” cells.^30^ Further studies confirmed these findings and reported that the number of injected tumor cells required for transplantation was further reduced by direct injection of cells into tumors exposed to a lethal dose of radiation one day prior to transplantation.^31^ Several studies have reported that more (often substantially more) than a thousand human tumor cells including human melanoma cells is required for the initiation of tumors in non-obese diabetic/severe combined immunodeficient (NOD/SCID) mice^32–34^. However as few as 1/9 human melanoma cells were capable of initiating tumors when injected with growth factor rich matrigel into NOD/SCID Il2rg-/- mice.^35^ In summary, substantial studies suggest that not all tumor cells are capable of initiating or sustaining tumors, and the fraction, even within the same tumor, may vary, depending on the tumor microenvironment and changes in the microenvironment attendant with tumor growth or response to therapy. Thus, it cannot be assumed that any tumor cell is incapable of sustaining tumor growth or giving rise to recurrence.

## Does conventional fractionated radiotherapy select for radioresistant cancer stem-like cells?

**Figure 1.**
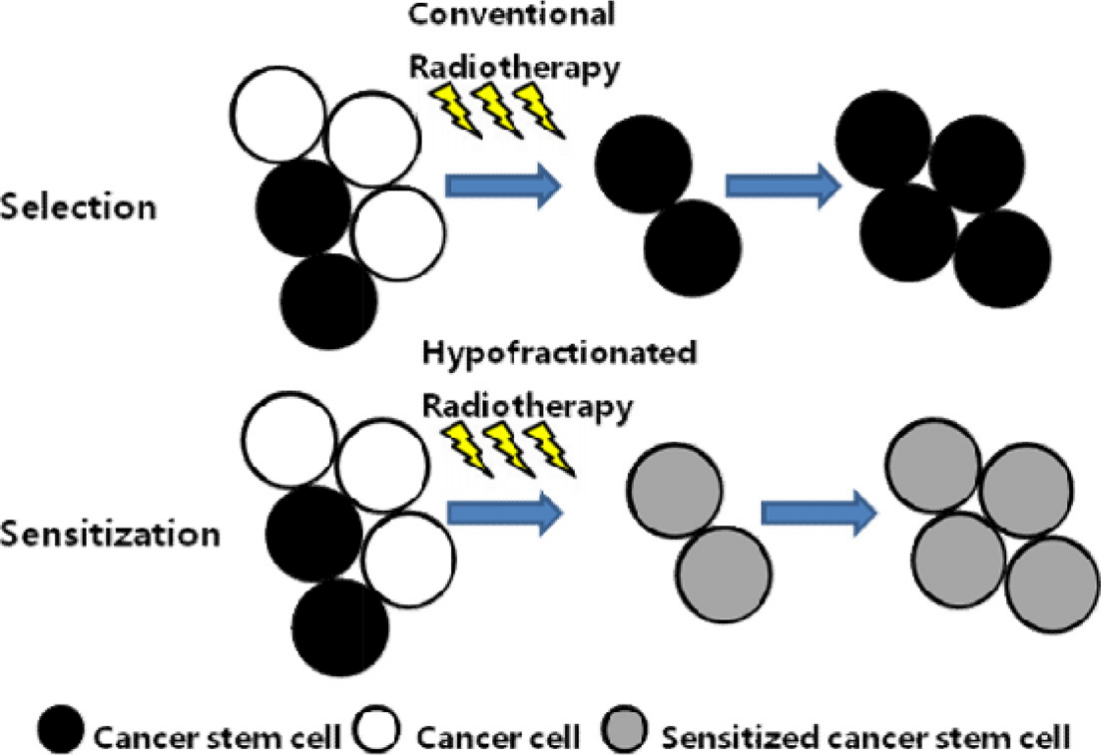
Current small dose per fraction radiotherapy may select for radioresistant stem-like cells while hypofractionated radiation may sensitize tumor cells to subsequent irradiation.

Treatment of recurrent tumors after conventional radiotherapy is more difficult than that of primary tumors, due to the reduced dose tolerance of previously exposed normal tissue. The treatment planning is further challenged by an assumed increased resistance of the recurrent tumor to conventional therapy compared to the primary tumor.^36^ Due to intratumoral heterogeneity, as previously noted, each small dose of radiation likely sterilizes a larger fraction of relatively radiosensitive cells, whereas the more radioresistant and presumably stem-like cells survive, leading to local recurrence as illustrated in Fig. 1. For example, Bao et al. showed radiation treatment enriched the CD133+ subpopulation in glioma, which was radioresistant due to activation of the DNA damage checkpoint response, resulting in a growth of DNA repair capacity. Furthermore, they reported a specific inhibitor of Chk1 and Chk2 reversed the cells’ radioresistance in vitro and *in vivo*, which provides a possible treatment option to reduce treatment failure.^37^ Mihatsch et al. showed that radioresistant cells were enriched from bulk lung cancer cells and breast cancer cells by multiple small doses of radiation, and that aldehyde dehydrogenase 1 (ALDH1) was a specific marker for the radioresistant cells.^38^ McDermott et al. showed multiple 2 Gy doses of radiation (60 Gy total dose) selected for radioresistant prostate cancer cells, which were less sensitive to DNA damage, and exhibited increased migration capacity. However, these radioresistant prostate cancer cells were more sensitive to docetaxel.^39^ Desai et al. found an increase in CD133+ cells following a single 4 Gy treatment in A549 lung cancer cells but not in H1299 lung cancer cells. The CD133+ enriched subpopulation exhibited radiation resistance.^40^ Zhang et al. established two radioresistant cell lines by exposing lung cancer cell lines H460 and A549 to 2 Gy/fraction once a week to a total dose of 60 Gy. They identified coxsackie-adenovirus receptor (CAR) as a new cancer stem cell marker.^41^ Shimura et al. found that hyperfractioned irradiation (0.5 Gy of X-rays every 12 h for 82 days) enriched the surviving cell population with CD133+ radioresistant cells in the hepatocellular carcinoma cell line HepG2 and glioblastoma cell line A172. These CD133+ radioresistant cells had higher tumorigenic capacity in nude mice. Compared with the radiosensitive parental cells, the AKT/cyclin D1/Cdk4 pathway was activated in CD133+ radioresistant cells. Specific AKT inhibitor API-2 or cyclin D1 siRNA sensitized the CD133+ radioresistant cells, which might be an efficient and safe method for the treatment of radioresistance.^42^

A second proposed mechanism of tumor radioresistance is the direct induction of cellular resistance by irradiation, rather than the selection of pre-existing resistant cells. That is, radiation induces the intracellular up-regulation of resistance factors and possibly the concomitant up-expression of tumor stem cell-like markers. Nielsen et al. found that after 12 fractions of 5 Gy, surviving murine Ehrlich ascites tumor cells (EHR2) over-expressed P-glycoprotein and multidrug resistance protein 1 (MRP1).^43^ Ionizing radiation has been proved to induce CSCs properties, including dedifferentiation and self-renewal.^44^ Cho et al. found that irradiated prostate cancer cell lines increased in CD44+ cell population that exhibit CSCs properties with long-term recovery.^45^ Dahan et al. reported that following a clinically relevant radiation dose, differentiated glioblastoma cells acquired a stem-like phenotype via survivin mediated increased expression of stem cell markers, and increased tumorigenicity.^46^

However, not all studies have observed either the radiation selection of resistant stem cells in the bulk tumor cell population, or the induction of resistance in previously sensitive cells. McCord et al. observed that CD133+ glioblastoma stem-like cells were sensitive to irradiation compared with current established cell lines (most established cells are CD133 negative).^47^ Dittfeld et al. found that CD133 expressing cells did not identify radioresistant subpopulations in colorectal cancer cells HCT116.^48^ Thus, although many previous studies reported fractionated small dose irradiation (usually < 5 Gy/fraction) resulted in accumulation of marker identified resistant stem-like cells that may give rise to tumor recurrence and metastases, these results are not consistently observed.

## Are tumor cells surviving large hypofractionated radiation radioresistant or radiosensitive?

Hypofractionated radiation therapy has shown possible advantages over conventional fractionated radiation (approximately 2 Gy/fraction) for some tumor types and is being explored or considered in additional types. Stereotactic radiosurgery (SRS) and SBRT are the two most commonly used hypofractionated radiation therapy schedules. SBRT is now the standard treatment for inoperable early-stage non-small cell lung cancer (NSCLC) or for those patients who refuse surgery, and is also feasible for operable cases recently.^49–51^ In patients with inoperable peripherally located stage I NSCLC, compared with conventional fractionated radiotherapy (66 Gy in 33 fractions or 50 Gy in 20 fractions), SBRT (54 Gy in 3 fractions, or 48 Gy in 4 fractions) brought favorable local control without an increase in toxicity.^50^ SBRT is an emerging primary treatment approach in clinically localized prostate cancer and has potential advantages over traditional radiotherapies.^52^ Following 7 Gy/fraction × 5 fractions or 7.25 Gy/fraction × 5 fractions, 5-year biochemical recurrence-free survival (RFS) was 97% for low risk and 74.1% for high-risk cancer, and adverse events were limited.^53^ The 5-year biochemical RFS was modestly better than conventionally fractionated radiation therapy (total doses of 86.4 Gy, up to 10 weeks of treatment).^53^ More importantly, the shorter treatment time increases the patient's life quality and preserves medical resources.

Treatment of tumors to an equivalent response metric, e.g. duration of progression free survival, can in principle be achieved by administration of multiple small doses of radiation or a single large dose. If the effect is subcurative, the question can be asked whether the initial treatment schedule, i.e. multiple small doses vs. a single or few large doses, impacts the total dose required to achieve permanent local control with a similar normal tissue complication probability or severity. Single large doses may damage or spare tumor vasculature vs. multiple small doses (typically a much larger total dose). The same damage or sparing effect may pertain to the remaining tumor cells. Experimental studies have suggested that achieving the same partial tumor response via different treatment schedules, may in fact influence the dose required to achieve permanent local control in recurrent tumors.^10,11^

In 1966, Suit reported that spontaneous murine tumors surviving a large single dose of radiation were more sensitive to subsequent irradiation than untreated tumors. A spontaneous murine mammary tumor was treated with a TCD95 radiation dose (resulting in local control of 95% of treated tumors). The recurrent tumor was excised and re-transplanted into syngeneic host mice. In contrast to expectations, the TCD50 was 51.3 Gy in the re-transplanted recurrent tumor but 59.9 Gy in the original tumor.^10^ The initial TCD95 radiation dose rendered the recurrent tumor sensitive to subsequent radiotherapy. Similarly, Ando et al. treated a murine fibrosarcoma by acute single dose radiation. The TCD50 was 58 Gy in previously hypofractionation treated tumor but 78.9 Gy in the original fibrosarcomas.^54^ Similar findings were reported by Majima et al., who found that after large subcurative doses of radiation, recurrent murine tumors were not more radioresistant than the original tumor.^11^ While these studies showed that recurrent tumors were equally or more sensitive than the original tumor, they did not resolve whether a change in tumor sensitivity was due to differences in the stem cell fraction of the original and recurrent tumor, or, whether differences in the oxygenation status could account for the differences in sensitivity. A possible explanation for these results is suggested by even earlier studies by Sinclair in 1964 (Fig. 1). Sinclair treated Chinese hamster ovary cells with large single radiation doses and observed that the radiosensitivity of the pretreated cells was 30% to 40% greater than that of the parental cells.^55^

Taken together, these studies defy the conventional expectation that cells surviving large doses of radiation are relatively radioresistant. Nevertheless, the reports are intriguing and warrant additional investigation. This is especially true in the era of precision medicine. As previously noted, with the increasing conformality of cancer treatment, e.g. particle therapy and IMRT, the possibility of treating and retreating recurrences while sparing normal tissue and thus normal tissue morbidity, is increasingly achievable. This is especially true for particle therapy, e.g. carbon and proton therapy. In contrast to X-irradiation, particle beams have an energy dependent finite range, thus sparing all normal tissue beyond the target volume. This results in a major reduction in normal tissue exposure.

## Mechanisms of radiation resistance

**Figure 2.**
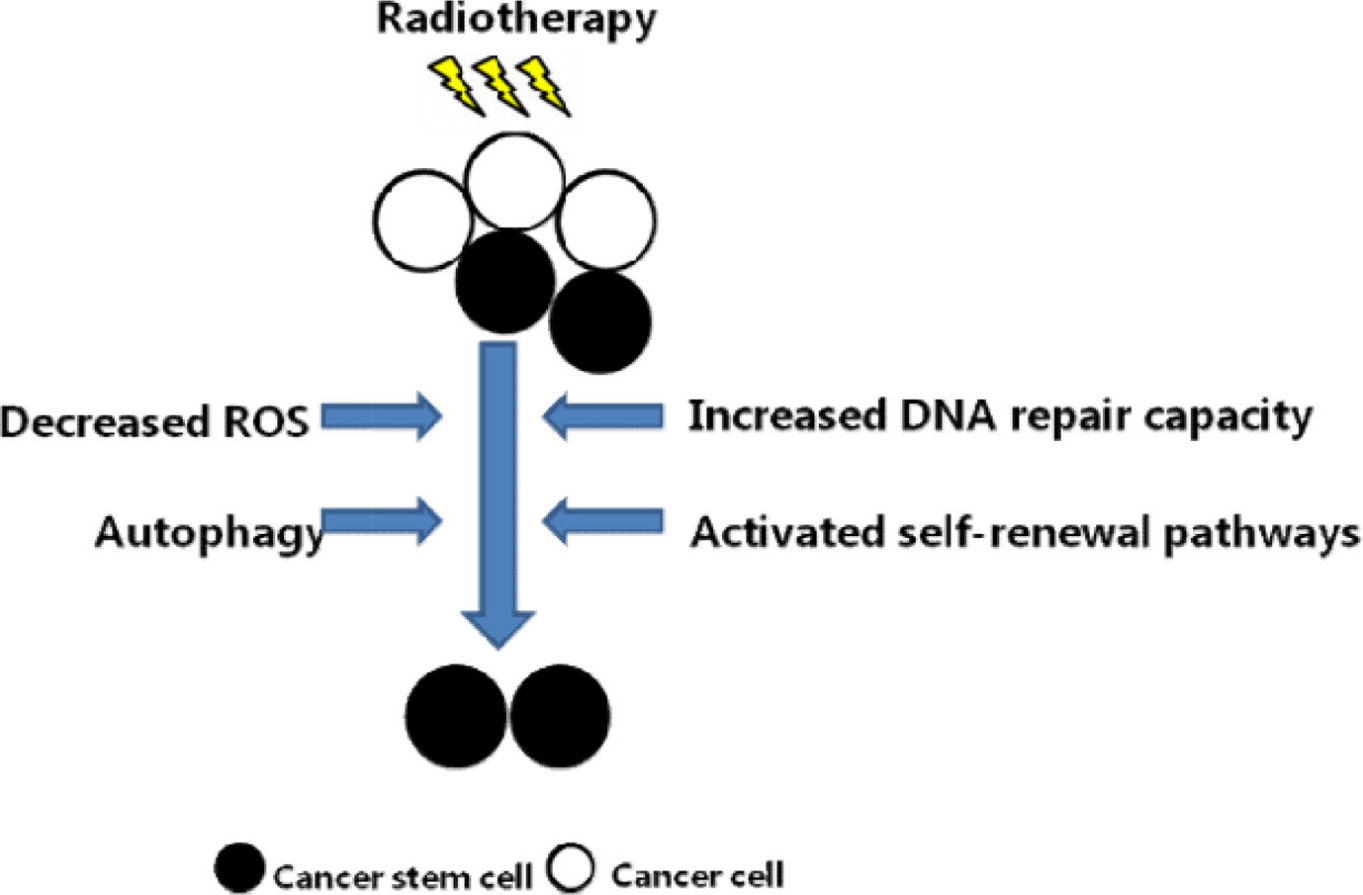
Proposed molecular mechanisms underlying cancer cell radioresistance. The contribution of each mechanism to resistance continues to be investigated.

As previously noted, tumor cells within the same tumor exhibit a range of sensitivities to radiation. The molecular mechanism underlying the reported radioresistance of stem-like cells is unclear. Studies have reported that radioresistance was associated with increased DNA repair capacity, activated self-renewal pathways, reduced reactive oxygen species (ROS), and autophagy (Table 1 and Fig. 2). Some other mechanisms of radioresistance of cancers include resistance to apoptosis, which makes modulation of apoptosis signaling pathways an important target for improving cancer therapy.^56^ Moreover, the roles of angiogenesis and immune microenvironment also had effects on radiation resistance.

**Table 1.** Pathways involved with radioresistance.

Pathway	Targeted molecular	Treatment	Reference
DNA DSB repair pathways			
HR	Chk1/Chk2	Solid tumors	^57,58^
	RAD51	Solid tumors	^59^
	PARP	Ovarian cancer	^60–62^
NHEJ	DNA-PKcs	Solid or hematologic malignancies	^63,64^
NOTCH signaling	Gamma-secretase inhibitor	Solid tumors	^65–67^
	DLL3/4	Solid tumors	^68,69^
Wnt/β-Catenin signaling	β-Catenin	Pancreatic adenocarcinoma	NCT01764477
	DKK1	Esophageal cancer or gastroesophageal junction tumors	NCT02013154
Sonic Hedgehog pathway	SMO	Solid tumors	^70–72^
EGFR/PI3K/Akt/mTOR pathway	PI3K/mTOR	Prostate cancer	^73^
	EGFR	Head and neck cancer	^74^

Abbreviation: DSB, double-strand breaks; HR, homologous recombination; NHEJ, nonhomologous end joining; SMO, smoothened; PARP, poly (ADP-ribose) polymerase; EGFR, epidermal growth factor receptor.

### Increased DNA repair capacity

DNA double-strand breaks (DSBs) are the most lethal lesion induced by ionizing radiation, if not repaired. Homologous recombination (HR) and nonhomologous end joining (NHEJ) are the predominant DNA DSB repair pathways.^75^ The HR pathway utilizes an undamaged DNA template to repair damaged sites, leading to more accurate DNA double-strand break repair than NHEJ. The HR repair functions during the mid S through the G2/M phases of the cell cycle and is less rapid than NHEJ.^76^ In contrast, NHEJ repair does not consider sequence homology and ligates broken ends directly, which can result in genome deletions or insertions. The NHEJ repair pathway responds immediately to a DSB, and functions throughout the cell cycle.^77^

It was known that CSCs showed altered DNA damage response and repair pathways comparable to tissue stem cells.^78^ The aberrant activation or increased expression of the NHEJ and HR pathways likely play an important role in tumor radioresistance. It was reported that CD44+/CD24– breast cancer stem cells and CD133+ glioma stem cells exhibited radioresistance and enhanced DNA repair capacity compared to non-stem cells through upregulation of the DNA damage-associated key proteins including ATM, Chk1/Chk2, ATR, and DNA-PKcs.^37,79,80^ Gene microarrays have also shown a close association between the increased expression of DNA repair genes and increased radioresistance in Lin-CD29+CD24+ breast cancer stem cells.^81^ Furthermore, post-translational modifications including ubiquitination, acetylation, methylation and SUMOylation may lead to the aberrant activation of the NHEJ pathway. For example, the ubiquitin ligase SPOP (Speckle-type POZ protein) may modulate DSB in prostate cancer. Mutated SPOP promotes prostate tumorigenesis through genomic instability and increases the response to DNA-damaging therapeutics.^82,83^ Lysine deacetylase SIRT1 may control the activity of several important DSB repair proteins including Ku70, NBS1, WRN and XPC.^84–87^ Several histone methyltransferases/demethylases, as well as their targeted histone methylations, participate in DNA-damage response.^88,89^ SUMO regulates DSB repair by affecting KDM5B/JARID1B and KDM5C/JARID1C.^90^

Since NHEJ and HR are the predominant DSB repair pathways, several studies have been devoted to the design of specific small molecule inhibitors targeting key proteins in the two pathways, such as ATM, ATR, Chk1/Chk2, poly (ADP-ribose) polymerase (PARP), and RAD51 inhibitors.^78^ Chk1/Chk2 inhibitors could act as sensitizers to radiation and DNA-damaging drugs, such as irinotecan and gemcitabine, and enhanced response in mouse tumor models.^91–93^ Some phase I clinical trials have explored the tolerability of these inhibitors with or without chemotherapy.^57,58^ However, further clinical trials are needed to verify their clinical benefits.

The RAD51 inhibitor amuvatinib sensitizes tumor cells to radio/chemotherapy by inhibiting RAD51 and HR in vitro and in vivo.^94^ A phase 1B clinical study showed amuvatinib was well tolerated and exhibited antitumor activity when combined with chemotherapy in neuroendocrine tumors and lung cancer.^59^ Clinical trials showed that PARP inhibitors, including olaparib, niraparib, and rucaparib, provided promising clinical benefits compared with traditional chemo/radiotherapy alone in BRCA1 or BRCA2 mutated ovarian cancer patients.^60–62^

In addition to the HR pathway, researchers have also focused on NHEJ pathway associated key proteins including DNA-PKcs which is an essential component of NHEJ. NU7026 and NU7441 were two of the most widely studied DNA-PKcs inhibitors and have been preclinically used to suppress the phosphorylation of DNA-PKcs and sensitize several tumor models to radiation.^95–97^ However, only one pharmacological inhibitor of DNA-PKcs (CC-115: a dual DNA-PKcs/mTOR inhibitor) has been assessed clinically, but not for the purpose of enhancing radiation sensitivity. Preliminary clinical data showed that CC-115, when used to treat chronic lymphocytic leukemia patients, decreased lymphadenopathy in 7 of 8 patients.^63^ Another phase I study confirmed well-tolerance and preliminary efficacy of CC-115 in advanced malignancy.^64^ Taken together, targeting the HR or NHEJ pathways appears to be promising in sensitizing tumors to radiation, and is being investigated in phase I-II clinical trials. However as with all toxic agents and sensitizers, an increase in the therapeutic ratio will be dependent on a differential effect on tumor versus normal tissue.

### Activated self-renewal pathways

Many developmental pathways (including NOTCH, Wnt/β-Catenin, Sonic Hedgehog, EGFR/PI3K/Akt/mTOR pathways) that maintain the self-renewal property of cancer stem-like cells have been identified. The aberrant activation of these pathways is associated with radioresistance.^98^ Inhibiting key proteins of these pathways can sensitize tumor cells to radiation.

NOTCH signaling is necessary for the maintenance of stemness of both normal and cancer stem cells.^99,100^ It has been reported that NOTCH signaling is dysregulated in tumor cells, which leads to their proliferation, invasion and metastasis. For example, NOTCH pathway ligands (DLL1, DLL3), receptors (NOTCH1, NOTCH2) and target genes (HES1, HES5) were upregulated in gliomas compared with non-tumor brain tissues.^101^ NOTCH1 and NOTCH2 knockdown was shown to sensitize CD133+ glioma stem cells to radiation. In addition, γ-secretase inhibitors were reported to reduce the colony forming ability of CD133+ glioma stem cells, but not of CD133- cells.^102^ However, phase I-II clinical trials showed that inhibitors of NOTCH signaling, while moderately safe, have only minimal to moderate influence on tumor progression, although these studies were not performed in combination with radiation.^65–67^ Thus, the development and screening of more effective chemical compounds which can inhibit NOTCH signaling is warranted.

The Wnt/β-Catenin signaling pathway functions in self-renewal, dedifferentiation, apoptosis inhibition, and metastasis in cancer development.^103^ Previous studies showed that components of the Wnt/β-Catenin pathway were abnormally activated or mutated in different types of cancer, and Wnt/β-Catenin signaling is implicated in the radioresistance of diverse tumors.^104–106^ For example, Kim et al. found Wnt/β-Catenin signaling associated proteins were activated in radioresistant glioma cells. Knock-down of these proteins sensitized resistant glioma cells to radiation.^107^ In a study by Jun and colleagues,^108^ the underlying mechanisms of how Wnt/β-Catenin signaling mediated radioresistance was revealed. DNA ligase IV was identified as a direct target of β-Catenin, and Wnt signaling enhanced NHEJ repair was mediated by DNA ligase IV transactivated by β-Catenin. Inhibition of DNA ligase IV sensitized tumor cells to radiation. Furthermore, the Wnt/β-Catenin pathway has been proved to be involved in CSCs radioresistance by improving the levels of activated β-catenin and promoting the proliferation of CSCs and their stability after radiation.^109,110^ Until now, the Wnt/β-Catenin signaling pathway has provided many potential therapeutic targets for the development of new drugs, some of which showed substantial inhibitory effects on many types of mouse tumor models.^111–113^ Many small molecule inhibitors and monoclonal antibodies targeting the Wnt/β-Catenin pathway are being studied in early clinical trials (mostly phase I, with a few phase II trials).^114^ Based on the significance of Wnt/β-Catenin signaling in cancer biology, drugs targeting the Wnt pathway may achieve better anti-tumor effects compared to conventional chemotherapy.

The Sonic Hedgehog pathway, which mediates embryonic development and is inhibited in adults, also plays an important role in carcinogenesis, invasion, and metastasis.^115,116^ Activation of the Sonic Hedgehog pathway is found in many types of tumors.^117^ Furthermore, researchers also found that the Sonic Hedgehog pathway is implicated in DNA damage repair and its activation is reported to be a mechanism for resistance to radiation. Chen et al. reported that the Sonic Hedgehog pathway was activated in human hepatocellular carcinoma following ionizing radiation.^118^ Chaudary et al. showed that Sonic Hedgehog inhibition could enhance the efficacy of radiation in orthotopic cervical cancer xenografts.^119^ Gonnissen et al. showed that GANT61, a Hedgehog inhibitor, could sensitize prostate cancer cells to radiation.^120^ Considering the importance of the Sonic Hedgehog pathway in tumor development, many drugs targeting this pathway are being designed and studied. The most well-known are Smoothened (SMO) inhibitors, vismodegib and sonidegib, which were approved by the FDA for the treatment of metastatic and/or recurrent locally advanced basal cell carcinoma (BCC).^70–72^ Newer drugs targeting the Hedgehog pathway and sensitize tumor cells to chemo/radiation will likely be developed in the future.

The last key signaling pathway involved in tumor radioresistance is the EGFR/PI3K/Akt/mTOR pathway. As is well known, amplified or mutated epidermal growth factor receptor (EGFR) can promote carcinogenesis through the signaling of downstream proteins including PI3K, Akt, mTOR and others.^121^ There is evidence that the upregulation of the EGFR/PI3K/Akt/mTOR pathway leads to tumor radioresistance.^122,123^ Hambardzumyan et al. found that the EGFR/PI3K/Akt/mTOR pathway was highly upregulated in medulloblastoma following radiation, and small molecule inhibitors of Akt signaling sensitized medulloblastoma cells to radiation.^124^ Chang et al. showed that the radioresistance of prostate cancer was associated with activation of PI3K/Akt/mTOR signaling, and the combination of a dual PI3K/mTOR inhibitor (BEZ235) with radiotherapy could surmount radioresistance in the treatment of prostate cancer.^73^ The same inhibitor could also sensitize five endometrial cancer cell lines to RT.^125^ With encouraging data from animal experiments, several clinical trials using inhibitors or monoclonal antibodies targeting the EGFR/PI3K/Akt/mTOR pathway have been initiated. The EGFR monoclonal antibody cetuximab combined with radiotherapy significantly improved overall survival at 5 years compared to radiotherapy alone in patients with locoregionally advanced head and neck cancer.^74^ The targeting of multiple targets within the EGFR/PI3K/Akt/mTOR pathway is currently under development, which may reduce radioresistance and further improve the clinical prognosis of cancer patients.^126–130^

### Reduced ROS induced DNA damage

DNA damage results from direct and indirect actions of X-rays. A direct action is caused by the interaction of photons or ionizing charged particles with DNA, resulting in its ionization. High linear energy transfer (densely ionizing) sources, such as carbon ion beams, induce DNA double-strand breaks mainly through direct action. An indirect action is mainly caused by ROS, which is produced by the interaction between secondary electrons and water molecule. X-rays induced DNA damage is mainly caused by indirect action. ROS generated from water radiolysis during radiotherapy comprises hydroxyl radicals and other radicals. High level of hydroxyl radicals could enhance oxidative stress to disturb cancer cells integrity and induce DNA damage, resulting in cell death.^131^ Previous studies reported that radioresistant cancer stem cells exhibit increased ROS defenses and lower ROS levels. For example, Diehn et al. showed that compared with non-tumorigenic cells, lower ROS levels were observed in breast cancer stem cells, which lead to less DNA damage after irradiation. Pharmacological depletion of ROS scavengers sensitized breast cancer stem cells to radiation.^132^ Kim et al. found that ROS were reduced in CD13 bearing liver cancer stem cells, which promoted their survival.^133^ These results suggest that reduced ROS may play a critical role in the radioresistance of CSCs and specific inhibitors targeting ROS degradation may sensitize cancer cells to radiation.

### Autophagy

Autophagy, a self-proteolysis procedure in eukaryotic cells, is activated by the detrimental cellular environment, leading to the breakdown of intracellular components within lysosomes to offer an alternative energy source and thus maintain cell survival.^134^ Recent studies suggest that autophagy may play a role in tumor cell survival following radiation therapy. Autophagy is frequently activated in tumor cells treated with chemotherapy or radiotherapy. Inhibition of autophagy has been reported to sensitize tumor cells to radiation in mouse tumor models.^135,136^ In contrast, some papers reported that the induction of autophagy might be a way to strengthen the anticancer effects of radiotherapy by autophagic cell death.^137,138^ These conflicting effects might because a dual role of autophagy in cancer cells (cell death vs. a pro-survival response). Qualitative and quantitative studies will be needed to further define the exact role of autophagy in cancer cells and radiation sensitivity.

## Summary

- Cancer is a heterogeneous disease and current small dose fractionated radiotherapy may select for radioresistant stem-like cells, which, if not sterilized, leads to local recurrence and distant metastases.
- In contrast to conventional dose fractionation schedules which may spare radiation resistant tumor cells, acute large dose irradiation may sensitize radiation resistant tumor subpopulations.
- Due to the greater sparing of normal tissue, particle beam radiation may be exploited for the administration of large dose per fraction radiation, and the re-treatment of recurrent tumors.
- Radioresistance mechanisms appear to primarily be associated with increased DNA repair capacity; however, activated self-renewal pathways, reduced ROS induced DNA damage, and possibly autophagy, may also contribute to resistance. The relative extent to which each mechanism contributes to cell lethality requires further study.
- Small molecule inhibitors targeting radioresistance associated pathways may enhance the therapeutic efficacy of radiotherapy, with potentially broad clinical application.
